# Use of microRNAs as Diagnostic, Prognostic, and Therapeutic Tools for Glioblastoma

**DOI:** 10.3390/ijms25052464

**Published:** 2024-02-20

**Authors:** David Valle-Garcia, Verónica Pérez de la Cruz, Itamar Flores, Aleli Salazar, Benjamín Pineda, Karla F. Meza-Sosa

**Affiliations:** 1 Laboratorio de Neuroinmunología, Instituto Nacional de Neurología y Neurocirugía Manuel Velasco Suárez (INNNMVS), Mexico City 14269, Mexico; david.valle.edu@gmail.com (D.V.-G.); ifloresm1903@alumno.ipn.mx (I.F.); aleli.salazar@innn.edu.mx (A.S.); 2 Laboratorio de Neurobioquímica y Conducta, Instituto Nacional de Neurología y Neurocirugía Manuel Velasco Suárez (INNNMVS), Mexico City 14269, Mexico; veped@yahoo.com.mx

**Keywords:** non-coding RNAs, microRNAs, miRNAs, glioma, glioblastoma, glioblastoma multiforme, GBM, glioma treatment, RNA therapy

## Abstract

Glioblastoma (GB) is the most aggressive and common type of cancer within the central nervous system (CNS). Despite the vast knowledge of its physiopathology and histology, its etiology at the molecular level has not been completely understood. Thus, attaining a cure has not been possible yet and it remains one of the deadliest types of cancer. Usually, GB is diagnosed when some symptoms have already been presented by the patient. This diagnosis is commonly based on a physical exam and imaging studies, such as computed tomography (CT) and magnetic resonance imaging (MRI), together with or followed by a surgical biopsy. As these diagnostic procedures are very invasive and often result only in the confirmation of GB presence, it is necessary to develop less invasive diagnostic and prognostic tools that lead to earlier treatment to increase GB patients’ quality of life. Therefore, blood-based biomarkers (BBBs) represent excellent candidates in this context. microRNAs (miRNAs) are small, non-coding RNAs that have been demonstrated to be very stable in almost all body fluids, including saliva, serum, plasma, urine, cerebrospinal fluid (CFS), semen, and breast milk. In addition, serum-circulating and exosome-contained miRNAs have been successfully used to better classify subtypes of cancer at the molecular level and make better choices regarding the best treatment for specific cases. Moreover, as miRNAs regulate multiple target genes and can also act as tumor suppressors and oncogenes, they are involved in the appearance, progression, and even chemoresistance of most tumors. Thus, in this review, we discuss how dysregulated miRNAs in GB can be used as early diagnosis and prognosis biomarkers as well as molecular markers to subclassify GB cases and provide more personalized treatments, which may have a better response against GB. In addition, we discuss the therapeutic potential of miRNAs, the current challenges to their clinical application, and future directions in the field.

## 1. Glioblastoma

Gliomas are derived from glial cells. In addition to being the most lethal and representing 30% of all primary tumors in the adult central nervous system (CNS), gliomas also encompass 75–80% of all malignant tumors within the CNS [1,2]. Glioma hallmarks include a rapid growth rate, high invasion and metastasis capacities, and resistance to treatment. Based on their histological and immunobiological characteristics, gliomas have largely been classified into astrocytomas, brain stem gliomas, ependymomas, oligoastrocytomas (mixed gliomas), oligodendrogliomas, and glioblastomas [3]. In 2016, the World Health Organization (WHO) proposed a classification of brain tumors from grades I to IV, depending on whether tumors present nuclear atypia, microvascular proliferation, mitotic activity, and necrosis [1,3]. Gliomas then can be classified as low-grade (I and II) or high-grade (III and IV) based on their growth and invasion potential. More recent research has allowed the WHO to better categorize gliomas while considering not only histological but also molecular features [4]. By following this approach, prognostic and therapeutic benefits have been observed, particularly in cases in which histologically identical grade tumors have different responses to treatment and, therefore, distinct survival outcomes.

In adults, glioblastoma (GB), also referred to as grade IV astrocytoma or glioblastoma multiforme (GBM), represents the most aggressive and common type of cancer within the CNS, as it rapidly grows and invades the brain and the spinal cord [5,6]. Although many of the histological and functional hallmarks of GB have been described—as well as the participation of genetic factors such as genomic rearrangements and point mutations that lead to the inactivation or altered functioning of genes, such as isocitrate dehydrogenase 1 (*IDH1*) [1,3,7], canonical tumor suppressor gene tumor protein P53 (*TP53*) [7,8], phosphoinositide 3-kinase (*PI3K*)/protein kinase B (*AKT*)/mechanistic target of rapamycin kinase (*MTOR*) [7], phosphatase and tensin homolog (*PTEN*) [9], retinoblastoma (*RB*) [1,7,8,10], epidermal growth factor receptor (*EGFR*) [7,11], and many genes in their downstream signal transduction pathways—which have been largely reported, the truth is that less than 5% of GB cases are only explained by genetic syndromes, mutations, or single-nucleotide polymorphisms (SNPs) in these genes [1,2]. Thus, most of the nongenetic molecular mechanisms involved in the etiology and progression of most GB cases are scarcely known.

Within the nongenetic mechanisms of gene expression control, both epigenetics and non-coding RNAs (ncRNAs) have been described to play essential roles in both physiological and pathological conditions by modifying gene expression at different levels. Among them, the altered expression of a small class of ncRNAs known as microRNAs (miRNAs) has been reported to occur in every type of cancer, including GB [6,12]. Moreover, the involvement of miRNAs in providing cancer with its typical intra- and intertumoral heterogeneity has also been reported for these tumors. However, the roles of miRNAs in the etiology, chemo-resistance, immune-evasion capacity, and overall malignancy of GB are not fully understood. 

The need for novel diagnostic, prognostic, and therapeutic options for GB patients is pressing. With an average 2-year survival of only about 20% and a 5-year survival of less than 5–8%, it is one of the deadliest cancers [13]. One of the reasons for this poor prognosis may be related to the fact that diagnosis often occurs once the tumor has reached a significant size and the patient presents clear neurological symptoms, often because of a high grade of malignancy. Diagnosis typically involves the use of imaging techniques, such as positron emission tomography (PET) scans, computed tomography (CT) scans, and nuclear magnetic resonance (NMR), and, in addition, it often requires a biopsy for pathological confirmation. These are expensive and risky procedures and it is not surprising that they are only applied once the physician has a strong suspicion of brain cancer; thus, these methods are not feasible to use as preventive or early detection methods. Therefore, the use of less invasive biomarkers that can be acquired in a more accessible manner and are easy to study either from tissue or from plasma or sera would greatly enhance our ability to detect GB early. In this regard, miRNAs are excellent candidates.

## 2. miRNAs

**Figure 1:**
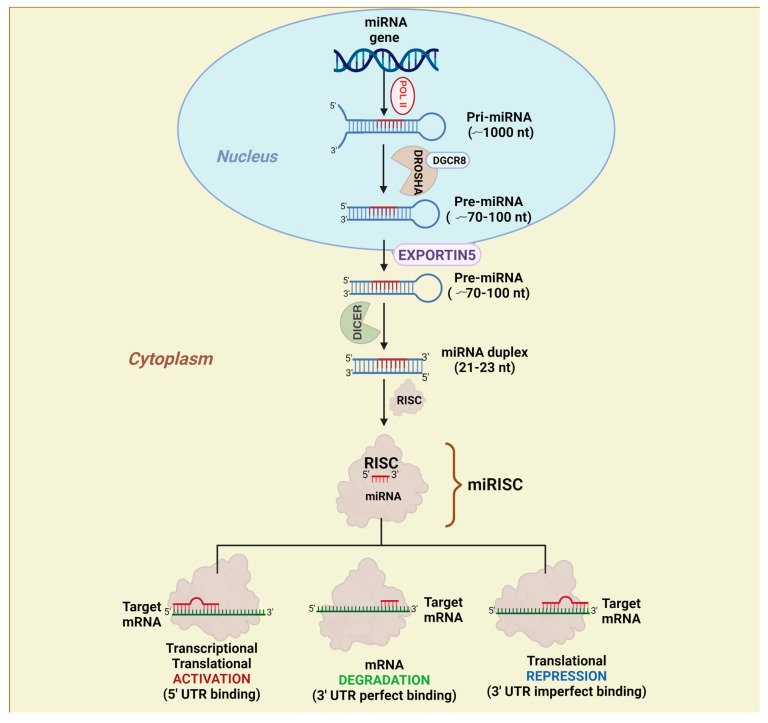
miRNA biogenesis pathway. miRNA biogenesis begins with the transcription of a miRNA gene by RNA polymerase II (POLII), which results in the production of a long transcript called primary miRNA (pri-miRNA) that is recognized by the microprocessor complex consisting of the type III RNAse, DROSHA, and the DiGeorge syndrome critical region gene 8 (DGCR8) protein. The pri-miRNA is then processed to generate a precursor miRNA (pre-miRNA) with a size of 70−100 nucleotides (nts). The pre-miRNA has a stem–loop structure and it is transported from the nucleus to the cytoplasm by the GTPase, EXPORTIN 5. Once in the cytosol, the loop region of the pre-miRNA is removed by another type III RNAse named DICER, resulting in the generation of an RNA duplex with an approximate size of 21–23 nt. One strand of the duplex will constitute the mature miRNA, while the other one will be degraded. Once the mature miRNA is released, it recruits the RNA-induced silencing complex (RISC) and, depending on the degree of sequence complementarity and the recognized region within the miRNA target messenger RNA (mRNA), gene expression will be either positively or negatively regulated by different mechanisms of action: transcriptional or translational induction (if the miRNA binds to the 5′ untranslated region (UTR) of the mRNA with imperfect sequence complementarity), mRNA degradation (if the miRNA binds to the 3′ UTR of the mRNA with perfect/almost perfect sequence complementarity), and translational repression (if the miRNA binds to the 3′ UTR of the mRNA with imperfect sequence complementarity).

miRNAs are small non-coding RNAs with a length of 21–23 nucleotides (nts) [14]. miRNAs are endogenous to organism genomes, and they are generated from a step-by-step biogenesis process (Figure 1). Firstly, miRNA genes are transcribed via RNA polymerase II (POLII) to produce a primary transcript (pri-miRNA) of around 1000 nts. Pri-miRNAs are then processed by the “microprocessor complex” consisting of the double-stranded RNA-binding protein DiGeorge syndrome critical region 8 (DGCR8) and RNAse III, DROSHA, giving rise to stem–loop precursor miRNAs (pre-miRNAs), which usually have an approximate size of 70–100 nts, a two-nucleotide overhang at their 3′ ends, and a 5′ phosphate group as a result of being processed via DROSHA [14]. The next step is guided by the GTP-dependent protein, Exportin-5, and, once in the cytoplasm, pre-miRNAs are recognized by another RNAse III called DICER. DICER removes the loop part of the pre-miRNA, and the resulting RNA duplex consists of the passenger RNA strand (previously known as miRNA*) and the future mature miRNA strand, which normally exerts negative control over gene expression. Then, this duplex is recognized by the RNA-induced silencing complex (RISC) and the miRISC complex is formed, depending on its sequence; the mature miRNA binds to complementary sequences found in the 3′ untranslated region (3′ UTR) of its target messenger RNAs (mRNAs). When the mature miRNA is almost fully complementary to its target sequence, the mRNA is degraded by a cut made by Argonaute (AGO). However, when only the miRNA’s seed sequence is complementary to its target mRNA, translational inhibition is promoted [15,16,17]. Although most miRNAs negatively regulate gene expression, either by mRNA degradation or translational inhibition, some cases of miRNAs that bind to the 5′ UTR of mRNAs have been reported to induce transcription [18,19] and mRNA stability and, thus, enhanced translation [20]. Therefore, gene expression control by miRNAs depends on both the degree of sequence complementary between miRNAs and their target sequences and the mRNAs’ region recognized by miRNAs [15,16,17,21]. 

miRNAs can act as either oncogenic (oncomiRs) or tumor-suppressing molecules depending on the cellular context. Thus, it is not surprising that altered miRNA expression profiles have been described for different cancer types, and GB is not the exception [1,5,22,23,24,25,26,27,28,29]. Moreover, as previously mentioned, a single miRNA can regulate the expression of many target genes and one gene can be targeted by multiple miRNAs [30], which results in intricate regulatory networks that can be different according to the analyzed condition [31]. It is also known that, depending on the cancer type, the same miRNA can not only promote or inhibit tumor growth but can also regulate different target genes in each condition. miR-7, for example, can be an oncomiR or a tumor suppressor gene, and it also regulates distinct genes in a cellular context-dependent manner [32,33,34,35]. Notably, miR-7 expression levels present dynamic changes both in normal CNS development [36,37,38,39] and in some CNS disorders [40,41], including GB [32,42,43,44,45,46,47,48], and, as these facts are true for miR-7, they have also been reported for most of the miRNAs involved in tumorigenesis. 

Thus, as miRNAs can act as oncogenic or tumor suppressor molecules, it is not surprising that aberrant miRNA expression profiles have been reported to occur in every type of cancer, including gliomas [5,22,26,29,49].

## 3. miRNAs Function in Glioblastoma

**Figure 2:**
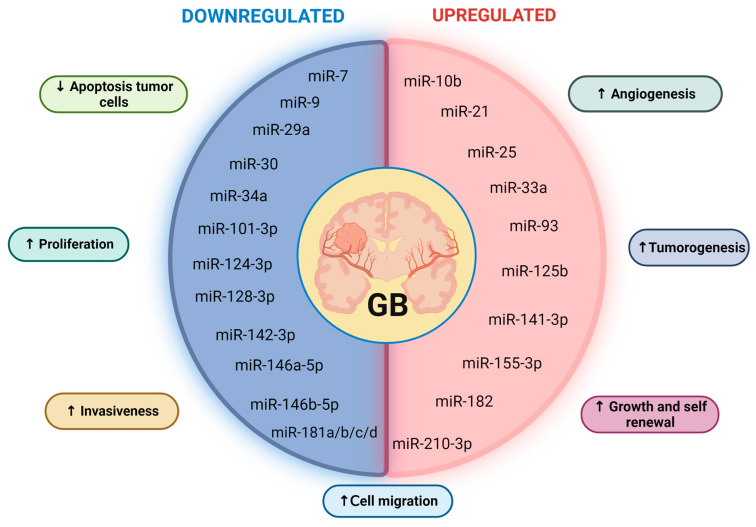
Downregulated (**left**) and upregulated (**right**) miRNAs in GB. Their related functions are depicted in boxes. Enhanced cellular functions are labeled with an upward-pointing arrow, while reduced cellular functions are labeled with a downward-pointing arrow.

Particularly in GB, the expression pattern of several miRNAs has been detected to be altered not only due to transcriptional defects or errors in their multistep biogenesis process but also due to genomic rearrangements, such as chromosomal translocations, insertions, and deletions involving genomic loci, in which, sometimes, there are miRNA genes [1,12,50]. As a result, several studies have applied genomics and deep sequencing techniques to analyze miRNA expression patterns in GB; actually, there is a vast number of miRNAs described to be either upregulated or downregulated mostly in in vitro models of GB, including several human and rat GB-derived cell lines [1,5,22,23,24,25,26,27,29,51]. This has been achieved by combining different experimental methods, including quantitative PCR (qPCR), microarrays, and deep sequencing. In this sense, miRNAs miR-9, miR-10b, miR-15a, miR-16, miR-17, miR-19a, miR-20a, miR-21, miR-25, miR-28, miR-93, miR-130b, miR-140, and miR-210 have been reported as upregulated in both GB cell lines and clinical samples of grade II gliomas progressing to grade IV secondary GB [5,52] (Figure 2). On the other hand, miR-7, miR-29b, miR-32, miR-34, miR-181 family members, miR-184, and miR-328 are among the downregulated miRNAs in GB progression [5,52] (Figure 2). In additional studies, some of these and other miRNAs have been detected to significantly change and directly influence GB hallmarks, such as proliferation, angiogenesis, immune evasion, resistance to cell death, metastasis, and appearance of chemo-resistance by the target genes they regulate in this context [1,6,12]. Here, we summarize the main role and some of the known target genes of miRNAs whose expression is significantly deregulated either in in vitro or GB tissue samples. Moreover, we discuss how these changes in both miRNAs and their target genes can influence GB progression and invasiveness. 

### 3.1. Upregulated miRNAs in Glioblastoma

#### 3.1.1. miR-10b

Usually, miR-10b has low expression in glial cells; however, in GB, it becomes overexpressed and it has been reported to negatively regulate the expression of genes involved in the control of cellular processes, including proliferation, migration, invasion, epithelial-to-mesenchymal transformation (EMT), and apoptosis. miR-10b exerts its oncogenic activity by directly targeting the epithelial cadherin 1 (*CDH1*) [53], apoptotic peptidase activating factor 1 (*APAF1*) [53], *PTEN* [53], *TP53* [54], patched 1 (*PTCH1*) [54], CYLD lysine 63 deubiquitinase or cylindromatosis (*CYLD*) [54], forkhead box O3 (*FOXO3*) [54], paired box 6 (*PAX6*) [54], homeobox D10 (*HOXD10*) [54], notch receptor 1 (*NOTCH1*) [54], BCL2-like 11 (*BCL2L11*) [55], transcription factor AP-2 gamma (*TFAP2C*) [55], cyclin-dependent kinase inhibitor 1A (*CDKN1A*) [55], and cyclin-dependent kinase inhibitor 2A (*CDKN2A*) [55] genes. Additionally, miR-10b was found to decrease cell sensitivity to radiotherapy-induced cell death [56]. Notably, its co-inhibition, together with that of miR-21, allows for its target genes to be expressed and, therefore, the suppression of GB tumor growth [57]. Thus, this miRNA is a promising molecule to develop future therapeutic approaches to stop GB progression.

#### 3.1.2. miR-21

miR-21 is deregulated in all described solid tumors, and GB is not the exception. miR-21 is considered an oncomiR, as its levels increase as tumorigenesis advances, and it downregulates the function of several cellular and molecular pathways by directly targeting genes such as the insulin-like growth factor (IGF)-binding protein-3 (*IGFBP3*) [58], reversion inducing cysteine-rich protein with kazal motifs (*RECK*) [59], and TIMP metallopeptidase inhibitor 3 (*TIMP3*) [59], resulting in enhanced cell migration and invasiveness and reduced apoptosis of tumor cells. Moreover, it was the first miRNA reported as being deregulated in this class of CNS tumor and its overexpression has been detected not only in brain tissue of GB patients but also in their cerebrospinal fluid (CSF), plasma, serum, and within serum- and CSF-derived exosomes [60], suggesting that this miRNA can be used as a less invasive diagnostic, prognostic, and even monitoring molecule of a patient’s response at different treatment stages.

#### 3.1.3. miR-25

miR-25 was corroborated to act as an oncomiR in GB cell lines as its overexpression promoted cell proliferation and invasion, while its inhibition caused the opposite effects. Moreover, miR-25 was shown to target the mRNA of the neurofilament light polypeptide (*NEFL*) gene directly [61]. In addition, miR-25 upregulation and *NEFL* downregulation were demonstrated to occur in human grade IV astrocytoma (GB) clinical specimens [61], suggesting an essential role for miR-25 in the development of GB malignancy and its potential as a therapeutic target for GB treatment. However, as discussed before, one miRNA can have a dual role in cancer progression, and, accordingly, miR-25 overexpression was shown to inhibit cell growth in an in vivo mouse model of GB by negatively regulating the expression of the p53 inhibitor mouse double minute 2 (*MDM2*) gene, thus promoting p53 accumulation in GB cells [51]. These point out that miR-25 is a positive regulator of p53, underscoring a new tumor suppressor role for it in GB tumorigenesis.

#### 3.1.4. miR-33a

The existence of cancer stem cells (CSCs) has been reported as an important characteristic of tumor growth, as these cells possess high proliferation and self-renewal rates [62]. In the context of GB, the presence of glioma-initiating cells (GICs) has also been demonstrated, and miR-33a expression promotes their growth and self-renewal by directly interacting with the mRNAs encoding phosphodiesterase 8A (*PDE8A*) and UV radiation-resistance-associated gene (*UVRAG*) genes, which are known to negatively regulate the cAMP/PKA and NOTCH pathways, respectively [63]. Notably, the activation of the NOTCH pathway commonly occurs in many cancers, as it promotes CSCs’ self-renewal and growth [64]. Thus, miR-33a-mediated reduction in *UVRAG* promotes the growth and self-renewal of GICs by enhancing NOTCH activity [63]. In this regard, the sole overexpression of miR-33a in non-GICs results in them acquiring GIC characteristics [63]. Moreover, it has been observed that elevated levels of miR-33a are associated with the poor prognosis of GB patients [63]. Thus, it seems that miR-33a has an oncogenic role in GB progression by promoting the activity of the NOTCH pathway. Additionally, miR-33a-5p was shown to negatively regulate the expression of the *PTEN* tumor suppressor gene in an in vitro model of GB [65]. Thus, in a more immunotherapeutic approach to treat GB, an inhibitor of the programmed death ligand 1 (PD-L1) has been demonstrated to enhance radiosensitivity of GB cancer cells not only by causing DNA damage but also by downregulating miR-33a-5p expression and, therefore, elevating PTEN levels [65].

#### 3.1.5. miR-93

miR-93 is a member of the miR-106b-25 oncogenic cluster of miRNAs. By itself, in vitro overexpression of miR-93 in GB-derived cells promotes cell survival, growth, and sphere formation capabilities, while, in an in vivo model of GB, miR-93 induces angiogenesis [66]. In addition, miR-93 negatively regulates the expression level of the integrin beta-8 (*ITGB8*) gene, which is an inducer of cancerous cell apoptosis [66]. Thus, increased miR-93 levels result in reduced apoptosis of tumor cells and, therefore, in enhanced GB growth [66]. Additionally, when miR-93-overexpressing GB cells are co-cultured with endothelial cells, the latter ones spread, grow, and migrate more [66], suggesting that miR-93 induces tumor growth, at least in part, by promoting angiogenesis.

#### 3.1.6. miR-125b

Despite miR-125b-2 being transcribed from a different genomic locus than miR-125b-1 (miR-125b), at the level of mature miRNAs, they share the same sequence and have the same regulatory effects within cells [67]. Thus, we will indistinctively call them miR-125b.

Usually, in the pro-neural subtype of GB, the activity of the canonical wingless-type (WNT)/β-catenin signaling pathway is exacerbated, leading to higher cell proliferation, increased formation of spheres, and inhibition of apoptosis [68]. Moreover, GB malignancy is enhanced as downregulation of its natural inhibitor, the frizzled class receptor 6 (*FZD6*) gene, is exerted by the GB-overexpressed miR-125b [69]. In addition, miR-125b was detected as being overexpressed in GB tissues and, in this same study, miR-125b inhibition in GB stem cells (GBSCs) enhanced the temozolomide (TMZ) apoptotic effect over them [70]. Even though direct interaction between miR-125b and the mRNA of the antiapoptotic B-cell CLL/lymphoma 2 (*BCL2*) gene was not validated, decreased BCL2 levels were observed when miR-125b was inhibited in GBSCs, suggesting that miR-125b overexpression might confer GBSCs’ resistance to TMZ by significantly reducing the levels of BCL2 [70].

It was also observed that the inhibition of miR-125b combined with the use of a PI3K inhibitor confers GBSCs’ enhanced resistance to TMZ through targeting the WNT/β-catenin signaling pathway [71]. Another study showed that oncogenic miR-125b confers TMZ resistance to GB cells by targeting both the TNF alpha-induced protein 3 (*TNFAIP3*) and the NF-κB inhibitor interacting Ras-like 2 (*NKIRAS2*) genes, as these cells also present increased NF-κB activity and upregulation of antiapoptotic and cell cycle genes [72]. In contrast, inhibiting miR-125b results in cell cycle arrest, increased apoptosis, and increased sensitivity to TMZ, indicating that endogenous miR-125b is sufficient to control these processes [72]. Most importantly, high levels of miR-125b were clearly associated with shorter overall survival of GB patients treated with TMZ [72], suggesting that this miRNA is an important predictor of patients’ response to treatment.

#### 3.1.7. miR-141-3p

Overexpression of miR-141-3p was observed in GB tissues compared to healthy brain. Moreover, a significant inverse correlation was also observed between miR-141-3p expression and P53 protein level [73]. After different experiments were performed in vitro, it was demonstrated that miR-141-3p directly targets the mRNA of the *TP53* gene and, therefore, it is not surprising that GB cells that present abnormal high levels of this miRNA proliferate more and present reduced cell cycle arrest and apoptosis. In addition, miR-141-3p overexpression also induces TMZ resistance of GB cells in vitro [73]. By using an orthotopic mouse model of human GB, inhibition of miR-141-3p reduced tumor growth within the brain and significantly increased mouse survival [73]. Thus, miR-141-3p might potentially serve as a new diagnostic marker and therapeutic target for GB treatment.

#### 3.1.8. miR-155-3p

miR-155 is one of the most studied miRNAs within the immune system and inflammatory processes. Thus, it is not surprising that alterations in its expression profile promote the occurrence of cancer-related processes, such as cell proliferation, cell cycle progression, apoptosis, and immune system evasion [74,75,76,77]. In the GB context, miR-155-3p has been shown to negatively regulate the expression of several genes related to tumorigenesis and the development of TMZ resistance, including Sine oculis homeobox homolog 1 (*SIX1*) [78] and protocadherin 7 (*PCDH7*) [55], the latter being a tumor suppressor that inhibits the WNT/β-catenin pathway. In this sense, miR-155-3p overexpression is oncogenic, as it induced cell proliferation and inhibited TMZ-induced apoptosis [78], and both oncogenic phenotypes were reversed by SIX1 overexpression and miR-155-3p inhibition. In addition, miR-155-3p inhibition reduced GB cell growth and proliferation in the brain of a mouse model and increased the survival of tumor-bearing mice [78]. Even though, in the context of GB, miR-155-3p target genes involved in regulating inflammatory pathways have not been extensively reported, it is feasible that this miRNA represents a good candidate for the development of future anti-GB RNA-based therapies [79,80,81,82,83,84,85,86].

#### 3.1.9. miR-182

miR-182 is an oncogenic miRNA that is positively regulated by different factors, such as the signal transducer and activator of transcription 3 (*STAT3*) [56] and the transforming growth factor beta (*TGF-β*) transcription factors (TFs) [87], which are aberrantly overexpressed in GB. Interestingly, miR-182 was shown to negatively regulate the protocadherin-8 (*PCDH8*) gene, which results in higher proliferation and invasion rates of GB cells [56]. In addition, miR-182 promotes a proinflammatory microenvironment within GB tumors by inhibiting the expression of the *CYLD* gene, a negative regulator of the nuclear factor kappa B (NF-κB) signaling pathway. Thus, as this miRNA is somehow related to the regulation of the immune response, it is possible that future GB therapeutic tools could be based on inhibiting the expression of this miRNA. In contrast, it has been observed that miR-182 sensitizes GB cells to TMZ treatment by inducing more apoptosis through the negative regulation of genes, including BCL2-like 12 (*BCL2L12*), MET proto-oncogene (*MET*), and hypoxia-inducible factor 2α (*HIF2A*) in a GB model of intracranial tumors [88]. Therefore, the modulation of miR-182 levels could also be used as an RNA-based therapy against GB.

#### 3.1.10. miR-210-3p

Hypoxia refers to a lack of oxygen and this typically occurs at the center of tumors, where regular vessels cannot supply it. In this sense, GB tumors present hypervascularization and necrosis, both caused by a hypoxic microenvironment. The hypoxia-inducible factor 1 subunit alpha (HIF1A) is the main transcription factor (TF) activated by hypoxia, which regulates the transcription of multiple target genes, including miRNAs. In this sense, miR-210-3p, together with miR-1275, miR-376c-3p, miR-23b-3p, miR-193a-3p, and miR-145-5p, were found to be upregulated by hypoxia in GB tissue, while miR-92b-3p, miR-20a-5p, miR-10b-5p, miR-181a-2-3p, and miR-185-5p were downregulated by hypoxia, and some of them present HIF1A binding sites within their promoter region [22]. Additionally, miR-210-3p was found to promote the hypoxic survival and chemo-resistance of GB cells by negatively regulating hypoxia-inducible factor 3 subunit alpha (*HIF3A*), a negative regulator of the of hypoxic response. In contrast, in the rat GB cell line C6, miR-210-3p acts as a tumor suppressor as it inhibits both cell proliferation and migration by negatively regulating the iron–sulfur cluster assembly enzyme (*Iscu*) gene [89], which makes the miR-210-3p/*Iscu* axis a potential target for the treatment of this type of glioma.

A summary of upregulated miRNAs and their targets is given in Table 1.

### 3.2. Downregulated miRNAs in Glioblastoma

#### 3.2.1. miR-7 

miR-7 is one of the most expressed miRNAs within the SNC. Some of its regular functions include the control of neural precursor proliferation and both neuronal and glial differentiation. miR-7 was reported to act as an oncomiR and as a tumor suppressor molecule, depending on the type of cancer. Moreover, miR-7 is downregulated in both tumoral tissue [29,44,49,90] and serum [25] of GB patients compared to healthy controls. Particularly, in GB, miR-7 was shown to act as a tumor suppressor miRNA, as it directly targets the epidermal growth factor receptor (*EGFR*) gene and negatively regulates the AKT signaling pathway through the direct inhibition of its upstream regulator, the insulin receptor substrate 2 (*IRS2*) [44]. Moreover, in this same study, the delivery of miR-7 into GB cell lines decreases both their viability and their invasion capacity [44]. In another study, miR-7 was shown to negatively regulate the T-box 2 (*TBX2*) gene, whose expression is upregulated in GB tissue; thus, miR-7 downregulation and TBX2 overexpression in GB results in both EMT induction and increased cell invasion of GB in vitro [90]. miR-7 was also shown to target the special at rich sequence binding protein 1 (*SATB1*) gene, the expression of which promotes GB cell migration and invasion [48], whereas focal adhesion kinase (*FAK*) downregulation by miR-7 results in decreased invasion of GB cells [47]. Notably, miR-7-5p suppresses stemness and enhances TMZ sensitivity of drug-resistant GB cells by targeting the yin and yang 1 (*YY1*) TF [43]. Moreover, in murine xenograft GB, miR-7 was capable of inhibiting tumor angiogenesis and growth by directly targeting the O-linked N-acetylglucosamine (GlcNAc) transferase (*OGT*) gene [32], and these same phenotypes were achieved when miR-7 targeted Raf-1 proto-oncogene (*RAF1*) in GB cell lines [45]. As observed, miR-7 regulates different hallmarks of GB progression, which makes this miRNA a very promising target for the development of future GB therapies. Additionally, a previous report showed that RNA-binding proteins quaking gene isoform 5 (QKI-5) and 6 (QKI-6) regulate the miR-7 biogenesis process [50]. Moreover, it was reported that pri-miR-7 processing to its mature miR-7 form is altered in GB cell lines [44]; however, the mechanism that regulates this process was not studied. Thus, it is possible that regulation by QKI-5 and QKI-6 represents one of the mechanisms by which miR-7 processing is controlled in GB, making it an additional target for future GB therapies.

#### 3.2.2. miR-9

The tumor suppressor miRNA known as miR-9 is typically downregulated in GB patient samples [91]. One of the mechanisms that downregulate miR-9 in the GB context is its epigenetic transcriptional silencing, which is mediated by enhancer of zeste homolog 2 (EZH2) [92]. In contrast to most miRNAs, both miR-9-5p and miR-9-3p (previously known as miR-9 and miR-9*, respectively) are expressed and functional [91], even though, in a contrasting study, miR-9 expression was found to be induced by hypoxia in GB cells [93]. Both miR-9-5p and miR-9-3p were shown to induce GBSCs’ proliferation by reducing the expression of the calmodulin-binding transcription activator 1 (*CAMTA1*) gene, which regularly induces the expression of antiproliferative cardiac hormone natriuretic peptide A (NPPA) [94]. In GB cells, miR-9 overexpression suppresses mesenchymal differentiation by downregulating the expression of Janus kinases 1 (*JAK1*), 2 (*JAK2*), and 3 (*JAK3*), resulting in the inhibition of the STAT3 signaling pathway [27], while it inhibits the proliferation of GB cells by targeting the cyclic AMP response element-binding protein (*CREB*) [95]. Moreover, the proliferation and aerobic glycolysis of GB cells are suppressed by miR-9 overexpression, which results in the downregulation of lactate dehydrogenase A (LDHA) [96]. In contrast to its regular tumor suppressor activity, miR-9 induces angiogenesis and cell migration by repressing sphingosine-1-phosphate receptor 1 (*S1PR1*) [92] and neurofibromin 1 (*NF1*) [95], respectively. Remarkably, a regulatory feedback loop is presented as CREB induces the transcription of both miR-9 and NF1. Moreover, miR-9 influences GB cells’ proliferation by regulating stathmin 1 (*STMN1*) [97], which regulates microtubule dynamics during cell division. On the other hand, miR-9 overexpression caused reduced invasion and migration of GB cells by blocking the formation of the mitogen-activated protein kinase 14 (*MAPK14*)/MAPK-activated protein kinase 3 (*MAPKAP3*) (*MAPK14/MAPKAP3*) complex by negatively regulating both genes and causing changes in the regulation of the actin cytoskeleton [94]. Moreover, miR-9 overexpression promotes apoptosis of GB cells by suppressing the expression of the structural maintenance of chromosomes 1A (*SMC1A*) gene in vitro [98]. Also, overexpression of miR-9 inhibits cell proliferation by directly targeting forkhead box P2 (*FOXP2*) [92]. Additionally, miR-9 is also involved in GB cells’ acquisition of chemo-resistance by negatively regulating the patched homolog 1 protein (*PTCH1*) gene. Additionally, the inhibition of miR-9 in resistant GB cells sensitizes them to chemotherapy [97]; however, most of its target genes during this process are currently unknown. In addition, sex-determining region y-box 2 (SOX2) TF, a direct target of miR-9-3p, is overexpressed in GB patients, leading to increased chemo-resistance, self-renewal, and tumorigenicity of GBSCs within GB tumors [97]. Thus, it is possible that the modulation of miR-9 levels represents a promising therapeutic strategy to diagnose and treat GB in the future.

#### 3.2.3. miR-29a

The miR-29 family of miRNAs has tumor suppressor functions, as they usually promote apoptosis of cancerous cells by targeting the cell division cycle 42 (*CDC42*) gene [99,100]. In GB tumors, miR-29 expression is usually decreased due to hypermethylation of its promoter region. Moreover, it was found that miR-29 overexpression inhibits proliferation, migration, and invasion of GB cells by negatively regulating the expression of genes, including the DNA methyltransferases 3 alpha (*DNMT3A*) and 3 beta (*DNMT3B*) [101], TNF receptor-associated factor 4 (*TRAF4*) [100], and *QKI-6* [102], with the latter interaction being a possible regulator of miR-7 levels in this context. As previously mentioned, GBSCs play an important role in GB progression; thus, the expression of genes in these cells is also crucial for tumor growth [103]. In this sense, it was found that miR-29a downregulation in GBSCs results in the overexpression of its target genes, platelet-derived growth factor subunit A (*PDGFA*) and C (*PDGFC*) [104], which leads to less apoptosis and higher proliferation, migration, and invasion rates of GB cells [100,104]. Thus, targeting miR-29a levels might be useful for GB treatment.

#### 3.2.4. miR-30a

miR-30a was found to be present in GB-cell-derived exosomes. Moreover, the overexpression of this miRNA increases GB cells’ chemosensitivity to TMZ by directly targeting Beclin 1 (*BECN1*) and by inhibiting autophagy [105]. Notably, it was demonstrated that miR-30a negatively regulates the expression of the brain-derived neurotrophic factor (*BDNF*) in a GB cell line when treated with paroxetine, a typical antidepressant drug [106]. Thus, even though the role of miR-30a:*BDNF* was not evaluated in the context of GB progression [106], it is possible that it has an important function in promoting tumor growth, as BDNF is known to induce cell survival.

#### 3.2.5. miR-34a

It is not surprising that miR-34a acts as a tumor suppressor miRNA, as it is a direct transcriptional target of P53. In GB tumors, *TP53* is one of the typically silenced/mutated genes and, therefore, its targets, including miR-34a, are also downregulated. In comparison to normal brain tissue, miR-34a is downregulated in GB and, accordingly, its overexpression in GB cells inhibits cell proliferation and induces apoptosis by directly inhibiting *BCL2* [28] and the nicotinamide adenine dinucleotide (NAD)+ hydrogen (NADH)-dependent sirtuin 1 (*SIRT1*) [28] genes. In an independent study, the platelet-derived growth factor (PDGF) signaling pathway was shown to repress miR-34a expression in GB. In this sense, the platelet-derived growth factor receptor alpha (*PDGFRA*) gene is directly targeted by miR-34a in this cancer type, thus constituting a negative feedback regulatory loop that results in GB progression [107]. In another study, it was shown that miR-34a directly targets different oncogenes in GB cells, including notch receptor 1 (*NOTCH1*) [108,109] and 2 (*NOTCH2*) [108] and cyclin-dependent kinase 6 (*CDK6*) [108]; therefore, miR-34a overexpression inhibits GB cells’ proliferation, cell cycle progression, survival, and cell invasion by targeting these three genes [108]. Moreover, miR-34a expression level is inversely correlated with Met proto-oncogene (*MET*) levels in human GB tumors [108]; however, whether MET is directly targeted by this miRNA was not evaluated. Another direct target of miR-34a that contributes to GB tumorigenesis by regulating translation is Musashi RNA-binding protein 1 (*MSI1*); thus, miR-34a overexpression reduced MSI1 protein levels, resulting in decreased cell proliferation [109]. The RPTOR-independent companion of the mTOR complex 2 (*RICTOR*) gene has also been reported as a direct target of a miR-34a and, through its downregulation, GB malignancy increases by indirectly activating both the AKT/mTOR and the WNT signaling pathways [28]. miR-34a was also demonstrated to directly target TF YY1, which results in EGFR overexpression and GB tumor growth [110]. Additionally, in p53-mutated GB tumors, the loss of miR-34a expression results in higher levels of its direct target gene, WNT ligand 6 (*WNT6*), which, in turn, activates WNT signaling and eventually promotes WNT-mediated GB chemo-resistance to TMZ. Thus, TMZ treatment, together with the inhibition of miR-34a, induces drug sensitivity in p53-mutant GB cells and extended survival in xenograft mice in vivo [111]. Noteworthy, by analyzing publicly available genomic data from GB patients, an integrative in silico study uncovered the potential regulation of the TGF-β signaling pathway, which is usually overexpressed in GB [87] via an SMAD family member 4 (SMAD4) transcriptional network, mainly orchestrated by miR-34a, which, at the same time, was observed to be a good discriminator of the pro-neural and mesenchymal GB subtypes [112]. These results indicate that using the already published, as well as future, bioinformatic analyses of available genomic data of GB patients [28] can provide a more comprehensive panorama of the intricate gene regulatory networks acting through GB progression to potentially enhance current and future diagnostic, prognostic, and therapeutic tools.

#### 3.2.6. miR-101-3p

miR-101-3p is considered a tumor-suppressor miRNA in many cancers [113]. It has many targets involved in cell proliferation and immune control and, thus, is downregulated in several cancer types [113]. In the context of glioblastoma, miR-101-3p is involved in inhibiting several tumor hallmarks, such as invasion, proliferation, migration [114,115], metastasis [116], and chemo-resistance [117]. miR-101-3p regulates many genes. It has been shown that, while the prostaglandin-endoperoxide synthase 2 (*PTGS2*) gene—which encodes for the COX2 protein—is upregulated in GB vs. normal tissue, miR-101-3p is markedly downregulated. PTGS2 regulates the conversion of arachidonic acid to prostaglandin (PGE2), which, in turn, enhances the activity of T-regulatory cells (T-regs), an anti-inflammatory cell type that reduces the immune response. miR-101-3p directly downregulates PTGS2 and drastically reduces invasion and proliferation [115]. On the other hand, miR-101-3p also inhibits GB proliferation, migration, and invasion in vitro as well as tumor growth in vivo, at least in part, by directly downregulating SRY-box transcription factor 9 (SOX9) TF, which, in turn, promotes the activity of the AKT, WNT, and BML1 pathways [114]. In addition, miR-101-3p downregulates the expression of the tripartite motif-containing 44 (TRIM44) gene. TRIM44 TF is a known regulator of the EMT process, and its inhibition by miR-101-3p reduces cell migration and proliferation [116]. Finally, it has also been shown that, in both TMZ-chemo-resistant GB cell lines and patient-derived samples, miR-101-3p is significantly downregulated. This chemo-resistant phenotype is reversed by miR-101-3p overexpression, and it is, in part, mediated by the downregulation of the glycogen synthase kinase 3 beta (*GSK3B*) gene that encodes for a protein kinase involved in cell metabolism.

#### 3.2.7. miR-124-3p

miR-124-3p is one of the most abundantly expressed miRNAs in neural tissue and it has an important function in neurodevelopment and neural cell differentiation [118]. Several studies have found that miR-124-3p is downregulated in GB, and this alters tumor cell growth, survival, migration, and chemo-resistance [119,120,121,122]. It has been shown that the mRNA of Ras homolog family member G (*RHOG*) is directly targeted by miR-124-3p in the GB context. RHOG is a small GTPase involved in cell migration. When miR-124-3p is downregulated, *RHOG* upregulation promotes cell proliferation and migration. Overexpression of miR-124-3p significantly decreases cell migration and survival and increases apoptosis levels [119]. miR-124-3p also downregulates SOS Ras/Rac guanine nucleotide exchange factor 1 (*SOS1*), which regulates the RAS/MAPK pathway by interacting with RAS and promoting guanosine diphosphate (GDP) to guanosine triphosphate (GTP) conversion to activate the pathway, resulting in enhanced cell survival. When miR-124-3p is downregulated in GB cells, *SOS1* expression is increased and it promotes cell growth [121]. Another notable miR-124-3p target gene is FOS-like 2, AP-1 transcription factor subunit (*FOSL2*), which gives origin to the Fos-related antigen-2 (FRA2) protein. *FOSL2* is a TF involved in EMT. *FOSL2* is upregulated in GB in response to miR-124-3p downregulation and its knockdown results in decreased cell proliferation, migration, and invasion [122]. miR-124-3p also negatively regulates the Aurora kinase A (*AURKA*) gene, a mitotic serine/threonine kinase involved in cell cycle control. *AURKA* is upregulated in GB, and its overexpression in patients is correlated with poor survival. *AURKA* overexpression enhances cell survival and chemo-resistance. In turn, *AURKA* downregulation by miR-124-3p overexpression suppresses cell growth and increases chemosensitivity [120]. 

#### 3.2.8. miR-128-3p

miR-128-3p is a tumor suppressor miRNA that is highly expressed in the mammalian brain. Thus, it is frequently downregulated in many cancer types, including GB [123]. In GB, miR-128-3p has been shown to regulate proliferation, migration, tumor formation, and chemo-resistance and may be involved in GBSCs biology [124,125,126]. miR-128-3p is particularly downregulated in GBSCs in comparison to regular tumor cells. Notably, treatment with DNA methylation inhibitors enhances miR-128-3p expression with higher effects in GBSCs, suggesting that miR-128-3p may be silenced by epigenetic mechanisms. Furthermore, miR-128-3p upregulation leads to decreased cell proliferation, migration, and invasion in vitro, and its overexpression decreases tumor growth in vivo. These effects seem to be mediated, at least in part, by the miR-128-3p-mediated downregulation of both the BMI1 proto-oncogene polycomb ring finger (*BMI1*) gene, which forms part of the epigenetic polycomb repressor complex 1 (PRC1), and the E2F transcription factor 3 (*E2F3*) gene, which is involved in the RB pathway [126]. Moreover, like miR-101-3p, miR-128-3p downregulates *PTGS2* expression to promote proliferation of GB cells [124]. Additionally, miR-128-3p is involved in GB cells’ acquisition of chemo-resistance. While miR-128-3p downregulation is correlated with high malignancy in vivo and in vitro, miR-128-3p overexpression has the opposite effect and it enhances chemosensitivity to TMZ treatment. In this context, miR-128-3p seems to downregulate both the platelet-derived growth factor receptor alpha (*PDGFRA*) and MET proto-oncogene, receptor tyrosine kinase (*MET*) genes, which are involved in EMT, thus enhancing the effect of TMZ. Therefore, the overexpression of MET abrogates this effect, whereas its silencing using small interfering RNAs (siRNAs) phenocopies miR-128-3p overexpression [125]. 

#### 3.2.9. miR-142-3p

miR-142-3p is downregulated in GB. It regulates proliferation, migration, invasion, and chemo-resistance and may have a considerable role in immunosuppression [127,128,129,130,131]. miR-142-3p regulates the EGRF pathway by directly inhibiting the AKT serine/threonine kinase 1 (*AKT1*) gene, thus decreasing cell proliferation when overexpressed [130]. It also mediates cell migration and invasion by directly downregulating Rac family small gtpase 1 (*RAC1*), a small GTPase involved in synaptic function and the regulation of matrix metalloproteinases [129]. miR-142-3p is also involved in regulating the immune response. Proinflammatory interleukin 6 (IL-6) promotes DNA methylation of miR-142-3p promoter, thus silencing it through epigenetic mechanisms. The *IL6* gene is a direct target of miR-142-3p, forming a regulatory loop. miR-142-3p also targets the high-mobility group at-hook 2 (*HMGA2*) gene, which is an activator of *SOX2* transcription, a well-known stemness marker. So, when miR-142-3p is downregulated, there is a high expression of both its direct target genes, *IL6* and *HMGA2*, and its indirect target gene, *SOX2*, which correlates with poor patient survival. Conversely, upregulating miR-142-3p in in vivo models decreases IL6, HMGA2, and SOX2 levels, leading to less tumor growth [128]. Noteworthy, it has also been found that miR-142-3p is markedly downregulated in tumor-infiltrating macrophages, which correlates with an M2 anti-inflammatory phenotype, although the precise mechanisms by which miR-142-3p may regulate this phenotype need to be further investigated [131]. Finally, miR-142-3p is also involved in chemo-resistance, as the O-6-methylguanine-DNA methyltransferase (*MGMT*) gene is its direct target. The MGMT protein acts in DNA repair by counteracting alkylating agents, such as TMZ, which is the most widely used chemotherapy agent against GB, an alkylating agent. Thus, miR-142-3p downregulation enhances *MGMT* expression and resistance to TMZ, whereas its overexpression reverts this effect [127].

#### 3.2.10. miR-146a-5p/miR-146b-5p

The miR-146 family of miRNAs comprises two members, miR-146a-5p and miR-146b-5p, which share an identical seed sequence and, therefore, possess similar targets. Both miRNAs arise from the same precursor and are negative regulators of the immune response. These miRNAs are highly expressed in microglia and other immune cells [132,133]. Although tightly linked, both miRNAs are usually studied individually and have distinct targets, although there is evidence that one can rescue the function of the other [132]. Both miR-146a-5p and miR-146b-5p are downregulated in GB and have targets involved in cell proliferation, migration, invasion, stemness, and chemo-resistance [134,135,136,137,138,139,140,141]. miR-146a-5p has been shown to directly downregulate *NOTCH1*, an important modulator of cell stemness and a pathway that enhances EGFR, one of the key gliomagenesis pathways. The knockdown of miR-146a-5p promotes tumorigenesis in astrocytes, while its overexpression in GB cells inhibits proliferation, migration, tumor growth, and GBSC formation, and it enhances apoptosis [134,136]. Moreover, miR-146a-5p negatively regulates POU class 3 homeobox 2 (*POU3F2*) and SWI/SNF-related, matrix-associated, actin-dependent regulators of chromatin, subfamily A, member 5 (*SMARCA5*), two TFs involved in stemness. Upregulation of miR-146a-5p reduces POU3F2 and SMARCA5 TFs levels and increases chemosensitivity to TMZ treatment. Furthermore, low levels of miR-146a-5p correlate with worse patient outcomes [135]. miR-146b-5p downregulates the EGFR pathway that is frequently upregulated in GB. Moreover, apart from epigenetic silencing, the locus that harbors both miR-146a/b is frequently lost in GB patients. Overexpression of miR-146b-5p reduces cell migration, invasion, and AKT phosphorylation, a hallmark of tumorigenesis [141]. Another target gene that is regulated by miR-146b-5p is TNF-receptor-associated factor 6 (*TRAF6*). TRAF6 is an adaptor protein that positively regulates both the PI3K/AKT and mitogen-activated protein kinase 7 (MAP3K7) pathways that enhance cell survival. miR-146b-5p expression is negatively correlated with both *TRAF6* expression and tumor grade in patient biopsies [140]. miR-146b-5p is also downregulated during the acquisition of TMZ chemo-resistance, while *TRAF6* expression is increased. miR-146b-5p overexpression reverts this phenotype [139]. Low miR-146b-5p levels, or its loss due to genomic rearrangements, are also correlated with increased migration and invasion capacities, at least partially through the regulation of the matrix metallopeptidase 16 (*MMP16*) gene, a matrix metalloproteinase. Upregulation of miR-146b-5p markedly reduced *MMP16* expression and GB cells’ migration and invasion capacities [137,138]. Although most reports have shown that the downregulation or loss of miR-146a/b indicates poor prognosis for patients [134,139,140], a recent report found that miR-146b-5p is upregulated in GB recurrence [142]. The reported cohort is small and further investigation is needed to assess whether miR-146b-5p can act as an oncogene in this context.

#### 3.2.11. miR-181a/b/c/d

The miR-181 family is a conserved family of four miRNAs that are produced by four genomic *loci*: one containing *miR-181a-1* and *miR-181b-1*, another one containing *miR-181a-2*, the third one containing *miR-181b-2*, and the last one containing the *miR-181c* and *miR-181d* genes. They all share the same seed sequence; thus, they may regulate a similar group of genes [143]. The whole family is downregulated in GB, and its downregulation positively correlates with tumor stage [144]. All miR-181 members have been found to negatively regulate the member of RAS oncogene family (*RAP1B*) gene, which is involved in cytoskeleton remodeling. Decreased *RAP1B* levels correlate with TMZ chemo-resistance, and the upregulation of miR-181a/b/c/d reverses this phenotype [145]. miR-181a-5p has been found to also downregulate F-box protein 11 (*FBXO11*) directly, which is part of the Skp, Cullin, and F-box (SCF) ubiquitin ligase complex that negatively regulates P53. In GB, low miR-181a-5p correlates with higher migration ability in cells. In contrast, its overexpression also correlates with lower migration and invasion rates, as well as with higher apoptosis rates, concomitant with lower *FBXO11* levels. Furthermore, miR-181a-5p overexpression increased TMZ chemosensitivity [146]. miR-181b-5p suppresses GB growth by inhibiting Sp1 transcription factor (*SP1*) TF. GB cells with low levels of miR-181b-5p have increased levels of SP1 and show increased levels of both the glucose transporter type 1 (*GLUT1*) and the pyruvate kinase M1/2 (*PKM2*) genes, which are regulators of glucose metabolism and are predicted targets of SP1 [147]. Furthermore, increased levels of miR-181b-5p decrease tumor growth in vivo, which is reversed by SP1 overexpression [147]. Lastly, it has been shown that miR-181d-5p directly downregulates *MGMT* expression (similar to miR-142-3p; see above). As expected, miR-181d-5p downregulation is correlated with poor patient survival when the patient underwent TMZ treatment but not when patients are untreated. This suggests that the miR-181d-5p-mediated regulation of *MGMT* affects TMZ chemo-resistance and low levels of miR-181d-5p would predict a poor drug response [148].

A summary of downregulated miRNAs and their targets is given in Table 2.

## 4. miRNAs as Biomarkers in Liquid Biopsies

Previously, we summarized some of the most representative miRNAs with a key role in GB’s appearance and progression. However, some of them have been detected not only in in vitro and postmortem GB samples but also in serum, plasma, and even circulating exosomes of GB patients [6]. This suggests that, as previously described for other types of cancer, circulating miRNAs are promising candidates to serve as early diagnosis and prognosis GB noninvasive biomarkers. Notably, it has been demonstrated that circulating miRNAs, independently of whether they are within exosomes or not, possess high stability and are practical and straightforward to detect in almost any kind of biofluids, including breast milk, blood, serum, saliva, urine, semen, and cerebrospinal fluid (CFS). Some of the reported circulating miRNAs in GB patients’ biofluids are listed in Table 3.

As well as being used as GB biomarkers, some miRNAs have been recently proven to serve as good monitoring molecules of GB patients’ response to treatment. Thus, the use of miRNAs for the development of future RNA-based GB therapies is very promising.

## 5. Modulation of miRNA Expression Levels as a Therapeutic Strategy in Glioblastoma

As mentioned before, it has been shown that many miRNAs are up- or downregulated in cancer mainly due to changes in the levels of both the TFs and epigenetic factors involved in the control of their transcription [24,159]. As mature miRNAs exert their regulatory function in the cytoplasm, it is key to understand that, before becoming functional to regulate gene expression, some of the steps of the canonical miRNAs’ biogenesis pathway (Figure 1) might be considered targets for the development of specific therapeutic tools. However, miRNA processing in GB has been scarcely studied and, therefore, there are no current strategies that have been developed to modify any step of this pathway. Moreover, as all the involved proteins in miRNAs’ biogenesis are responsible for the processing and generation of all cellular miRNAs, this means it is more complicated to develop tools to alter the production of a particular miRNA without altering the levels of many others. Thus, research in this area will be needed in the near future to first mechanistically explain cases as the one reported about the altered processing of pri-miR-7 to pre-miR-7 in GB [44] and, second, to develop therapeutic strategies to produce the desired amount of a particular mature miRNA.

Because cancer, in general, and GB, in particular, is a complex disease, it is to be expected that many genes may be deregulated and this naturally includes miRNAs [1,6,12]. However, there is mounting evidence showing that miRNA deregulation may be a causal factor for the disease and not a simple consequence of it. Such evidence has been shown both in in vitro and in vivo models (see above), and it is also suggested that distinct miRNA signatures can be found in large cohorts of patient samples [23,52,160]. In addition, many miRNAs have been shown to be part of key pathways that are involved in different aspects of GB biology, such as tumor formation, immune evasion, rapid growth, vascularization, cell diversity, formation of stem-cell-like cells, and chemo-resistance [24,159]. Moreover, miRNAs are recognized for their pleiotropic effects: one miRNA can modulate many targets and one target gene may be modulated by many miRNAs at the same time and in the same cell. This property has been proposed as a tool for finding key miRNAs in cancer that would have a significant effect on the transcriptional regulatory network if they are modulated [31]. 

Many strategies have been proposed to regulate mature miRNAs’ function, and two general classes of miRNAs can be identified in cancer. First, oncomiRs can be defined as miRNAs that promote tumorigenesis, cell growth, cell survival, immune evasion, etc. OncomiRs are often upregulated in GB (see the above; upregulated miRNAs in GB (Table 1)) [1,6,12,161]. As a therapeutic strategy, oncomiRs should be blocked or downregulated. On the other hand, tumor suppressor miRNAs are miRNAs that are involved in processes, such as differentiation, cell death, cell cycle control, immunogenicity, and other mechanisms, that inhibit tumor growth and formation (see the above; downregulated miRNAs in GB (Table 2)) [1,6,12,161]. Those miRNAs are downregulated in GB and overexpressing them would have a positive therapeutic effect. 

As mentioned before, a single miRNA can behave as an oncomiR or a tumor suppressor miRNA, depending on its cell context. It is not surprising that the same miRNA may be classified as oncogenic in one cancer type or subtype and as a tumor suppressor in another. Thus, finding good miRNA therapeutic targets involves a good understanding of the underlying biology and the effects that a particular miRNA has on its targets in a particular cell context [162,163]. Thus, many of the aforementioned findings may not be necessarily generalizable to all cell contexts (e.g., tumor stage, tumor type, whether the tumor is being treated or has been treated with chemotherapy agents, genetic ancestry of the patient, etc.), and it is likely that many therapies should be developed for specific tumor contexts. Therefore, more basic research needs to be carried out to enhance our understanding of miRNA (de)regulation in GB formation, growth, invasion, immune evasion, response to chemotherapy, etc.

That said, two general strategies can be followed to alter miRNA expression [162,163]. Because miRNAs are small molecules that, in principle, may cross the blood–brain barrier, synthetic mimics are often used to enhance their expression [161,164]. Like the RNA-based therapeutic approaches that have been used in COVID-19 vaccines, miRNA mimics can be delivered directly as double-stranded or single-stranded RNA molecules that would be directly loaded into the RISC (Figure 1). Those molecules often have modified nucleosides to enhance their stability. Alternatively, miRNAs can be delivered in vectors, either as complete genes including the sequence of the natural pre-miRNA or in synthetic miRNA-like precursors, which combine a known precursor with the sequence of the miRNA for which overexpression is desired. In both cases, the precursor is delivered as DNA and needs to be transcribed in the nucleus and processed as naturally occurring miRNAs. Both strategies pose advantages and disadvantages that are not dissimilar to those of COVID-19 mRNA/vector vaccines. 

When one wants to decrease miRNA expression, two popular strategies have been followed [162,163]. One is the use of antagomiRs as therapeutic tools. AntagomiRs (also called ASOs or antisense oligonucleotides) are RNA molecules with modified nucleosides that dramatically enhance their stability [165]. Those molecules are designed to be complementary to the miRNA sequence. Because of their enhanced stability, they can sequester miRNA molecules without being degraded. Because they are small molecules (virtually the same size as miRNAs), they are, in principle, easy to transport and deliver. The second strategy involves the use of miRNA sponges as a tool to decrease miRNA activity. Naturally occurring miRNA sponges are circular RNA molecules with dozens or even hundreds of miRNA binding sites [166]. As antagomiRs, miRNA sponges can also sequester miRNA molecules, allowing the expression of the original miRNA targets. The advantage of using sponges vs. antagomiRs is that one could potentially target several miRNAs with the same sponge if the target sequence of different miRNAs is included in the sponge sequence. One major disadvantage is that the generation of circular RNAs is technically more challenging than small single-stranded RNAs and their delivery may be more challenging depending on their size. 

Finally, with the advance of epigenetic editing tools, many of which are based on CRISPR/Cas systems, one may envision a future in which no external miRNA/anti-miRNA molecules need to be added [167]. Endogenous miRNA genes may be either silenced or overexpressed by modulating their epigenetic marks. One of the main advantages of such an approach is that the silencing or enhancing effect may be maintained by epigenetic mechanisms, potentially making the effects long-lasting and reducing the need for repeated doses. 

Independently of the method of choice, one of the main challenges for the realization of RNA-based therapeutics is the so-called delivery problem, which will be further discussed in the future of RNA-based therapies for GB section. 

## 6. Current FDA-Approved RNA-Based Therapies to Treat Cancer

COVID-19 was both a great challenge for humanity and clear proof of the power of RNA-based therapies. The quick development of many RNA-based vaccines, either vaccines based on mRNAs or vectors, has shown the great promise of RNA medicine. However, such a success would have been impossible to achieve without decades of research in RNA biology and therapeutics [168].

Although there are no currently approved RNA-based therapies to treat any type of cancer, this will very likely change in the near future.

In general terms, four types of RNA molecules have been approved for their use in therapeutics: mRNAs, ASOs, siRNAs, and aptamers [169,170]. Of these, siRNAs and ASOs are relevant for this review. Eight ASO-based therapies have been approved by the FDA so far, mainly for the treatment of rare genetic diseases. Fomivirsen was the first approved ASO therapy. It was approved in 1998 but it was quickly discontinued in 2002 due to secondary effects and risk concerns [169,170]. A second generation of ASOs was developed and Mipormersen was approved in 2013, followed by the successful Nusinersen in 2016, the first RNA-based approved therapy for Duchenne muscular dystrophy. Several ASO treatments, mainly for Duchenne and other muscular dystrophies, were approved between 2016 and 2021. Moreover, between 2018 and 2022, four siRNA-based therapies (which act very similar to endogenous miRNAs) were approved [169,170].

Although most of the approved therapies target very specific and somewhat rare diseases, the massive application of mRNA-based vaccines has dramatically increased our knowledge and the advancement of RNA-based technologies. This has boosted the interest in ongoing clinical trials as well as in starting new ones.

Several ASO-based therapies are being investigated for different cancer types in phase II clinical trials. Likewise, some siRNA-based therapies have been proposed, including the use of an inhibitor of Cbl proto-oncogene B (*CBLB*), which is being investigated in several cancers, including brain cancer, and it is currently in phase I [171]. Additionally, some miRNA-based strategies are being investigated for the treatment of other diseases, including miR-34a in the treatment of melanoma [171].

As is the case with many technologies based on nucleic acids and other molecular therapies, delivering the molecule to the correct place at the correct time is fundamental for therapeutic success and safety. Thus, investigating technologies for the efficient and safe release of RNA molecules will be key for the advancement of the field, together with a keen understanding of the (epi)genetic networks underlying disease states.

## 7. The Future of RNA-Based Therapies for Glioblastoma

Because the brain is generally isolated from the rest of the body through the blood–brain barrier, treating brain-related diseases has proved challenging in general [172]. This is not the exception with RNA-based therapies. Because miRNAs are molecules that exquisitely fine-tune gene expression in both time and space [118,173], it is to be expected that any therapy that modulates them also needs to be very time- and space-specific if one does not want to create unintended and potentially dangerous side effects [174]. The fact that, as discussed previously, the same miRNA can be both oncogenic or tumor-suppressing, depending on the cellular context, highlights the importance of achieving some grade of specificity on the target organ, preferably at the cell-type level [162,163].

Although it has been demonstrated that the blood–brain barrier becomes compromised in GB [175], it persists as a relevant barrier that has to be overcome to achieve sufficient molecule concentration for a therapeutic effect with minimal off-targets in other organs.

For this, two main strategies may be envisioned, which are not mutually exclusive. One is to develop small devices that can be surgically implanted in the brain near the tumor mass, which can release the RNA molecule (or a mix of RNA within lipid-based or another type of carrying particle as a cell delivery tool) either constantly or in fixed intervals [176,177]. Advanced models may even modulate the amount of RNA to be delivered based on other metrics that the same device could measure, such as the production of specific biomarkers, maybe even other miRNAs or other RNA types. Such a strategy has the advantage of potentially achieving a high concentration of the therapeutic RNA in the tumor while minimally affecting adjacent regions or other organs [176]. Moreover, the combination with other biomarkers and the possibility of fine-tuning or even personalizing the dose depending on the patient’s response would be ideal. However, besides the surgical risks inherent to such approaches, the device would likely be expensive and it would require highly trained personnel for installation, monitoring, and removal, thus making it inaccessible as the first type of treatment [176,177].

Another strategy is to develop a delivery method that allows higher specificity without the need of external hardware. Such strategies may use pseudo-viral or viral-like capsids, nanoparticles of various types, or lipid-based strategies. The advantages and disadvantages of such strategies have been reviewed extensively elsewhere [178,179] but we would like to point out a few of them. Strategies based on viral-like capsids take advantage of the natural tropism of several viruses. For example, the use of delivery systems based on the rabies virus, which has a high and specific brain tropism, has been proposed as an alternative to the challenge of reaching the brain without compromising other organs [180]. On the other hand, nanoparticles and nanotechnology, in general, are extremely promising fields. It is difficult to specify the characteristics of the technology, as each nanoparticle may have significantly different properties depending on its composition. However, in general, the goal would be to find a nanoparticle that allows for specific delivery to the brain, maybe even to the tumor, while avoiding releasing its cargo in other parts of the body. The material must be secure; it should not be immunogenic (at least not outside the context of the tumor), and, preferably, it should be relatively easy and cheap to fabricate. It is likely that such materials will be developed in the coming years as the field of nanotechnology advances quickly.

Much hope is put into the development of lipid-based particles [181]. The main advantage of lipid-based particles is that, as they are naturally generated and can have natural tropisms for specific organs and even particular cell types, they can achieve the goal of being specific, potent, and safe [181]. In fact, mRNA vaccines are delivered in lipid-based particles and, while they are not yet very specific, they have been proven to be safe [182]. Lipid-based particles can be generated artificially, which has the advantage of allowing a very homogeneous preparation, which is relatively easy to characterize, fabricate, store, and administrate [181]. Howevser, we still lack the knowledge to achieve extreme organ specificity with such types of particles. Naturally occurring extracellular vesicles, many of which contain RNA molecules, in particular miRNAs, may be an attractive alternative [183]. It has been shown, for example, in in vivo models of GB, that vesicles derived from irradiated cells can be used to enhance tumors’ immunogenicity [184]. Such strategies have the advantage of using cell mechanisms that, while they may not be fully understood, still achieve potent antitumor activity. The disadvantage is that their preparation may be complex and their molecular effects need to be explored before being used as therapies in humans.

Finally, CRISPR/Cas technologies may also provide new avenues for treatment and research. Because direct gene editing may be difficult to control and ethically controversial, epigenetic editing may be an attractive alternative [167]. Thus, the epigenetic editing of specific miRNAs may achieve considerable changes in the transcriptional network with only a few editions. The same delivery problems that apply to RNA molecules also apply to CRISPR/Cas systems, with the added complexity of delivering a relatively large protein (Cas). However, given the current research intensity in the field, it is likely that better and more potent CRISPR/Cas-based therapeutic strategies will be developed soon.

Although there are several roadblocks that need to be resolved, the use of RNA-based therapies, particularly those that use or modulate miRNAs, seems very promising for developing successful, potent, and secure therapies.

## 8. Conclusions

Despite focusing on highlighting miRNAs’ potential as diagnostic, prognostic, and therapeutic tools for GB, another class of non-coding RNAs longer than 200 nts—named long non-coding RNAs (lncRNAs)—has also been very useful in the study of cancer biology, including GB. Thus, it is not surprising that, by combining all knowledge regarding these two types of ncRNAs and future ncRNA-based therapies, GB biology and treatment might become more understandable and promising, respectively.

It is evident that more basic research is needed to increase our understanding of the relevant biology, particularly the effects that miRNAs have on the transcriptional network and how they are related to the remarkable ability of GB to grow aggressively while avoiding the immune system. Moreover, there is increasing information about the contributions of both the tumor microenvironment and the participation of immune cells, including monocytes/macrophages/microglia and T cells, in GB progression [7]. Therefore, targeting not only tumor cells but also blood vessels and immune cells should be considered in designing better clinical trials aimed to develop improved molecular-based treatments for GB. Such knowledge would allow us to discover diagnostic, prognostic, and therapeutic miRNAs to more effectively combat this devastating disease.

Finally, very little is known about the possible genomic variation in miRNAs between different populations. Although it would be desirable to find miRNAs that are always affected in GB independently of the context, perhaps a more realistic approach would be to consider the genetic and environmental differences that may affect the expression of specific miRNAs in different populations and even among different individuals. Thus, profiling both mRNA and miRNAs in populations with diverse genetic, ethnic, cultural, and environmental backgrounds would be key to enhancing our understanding of miRNAs and their application as diagnostic, prognostic, and therapeutic tools.

## Figures and Tables

**Table 1:** Upregulated miRNAs in GB.

miRNA	Target Genes	Carcinogenic Effect	References
miR-10b	*APAF1*, *BCL2L11*, *CDH1*, *CDKN1A*, *CDKN2A*, *CYLD*, *FOXO3*, *HOXD10*, *NOTCH1*, *PAX6*, *PTCH1*, *PTEN*, *TFAP2C*, *TP53*	Increased cell proliferation, migration, invasion, and EMT. Reduced cell sensitivity to radiotherapy-induced cell death.	[53,54,55]
miR-21	*IGFBP3*, *RECK*, *TIMP3*	Increased cell migration and invasion. Reduced apoptosis.	[58,59,60]
miR-25	*MDM2*, *NEFL*	Increased cell viability and proliferation. Reduced apoptosis.	[5,51,61]
miR-33a	*PDE8A*, *PDL1*, *PTEN*, *UVRAG*	Increased growth and self-renewal of glioma-initiating cells. Reduced patients’ survival rate.	[63,65]
miR-93	*ITGB8*	Increased cell survival, growth, sphere formation, and angiogenesis. Reduced apoptosis.	[66]
miR-125b	*BCL2*, *FZD6*, *NKIRAS2*, *TNFAIP3*	Increased cell proliferation, sphere formation, and chemo-resistance. Reduced apoptosis.	[69,71,72]
miR-141-3p	*TP53*	Increased cell proliferation and chemo-resistance. Reduced apoptosis.	[73]
miR-155-3p	*PCDH7*, *SIX1*	Increased cell proliferation, immune system evasion, and chemo-resistance. Reduced apoptosis.	[55,78]
miR-182	*BCL2L12*, *CYLD*, *HIF2A*, *MET*, *PCDH8*, *STAT3*	Increased cell proliferation, invasion, NF-κB-mediated inflammation, and chemo-resistance. Reduced apoptosis.	[56,87,88]
miR-210-3p	*HIF3A*, *Iscu*	Increased cell proliferation, migration, tumor hypoxia, and chemo-resistance.	[22,89]

**Table 2:** Downregulated miRNAs in GB.

miRNA	Target Genes	Carcinogenic Effect	References
miR-7	*EGFR*, *FAK*, *IRS2*, *OGT*, *RAF1*, *SATB1*, *TBX2*, *YY1*	Increased cell viability, migration, invasion, angiogenesis, and EMT.	[32,43,44,45,47,48,90]
miR-9	*CAMTA1*, *CREB*, *FOXP2*, *JAK1*, *JAK2*, *JAK3*, *LDHA*, *MAPK14*, *MAPKAP3*, *NF1*, *PTCH1*, *S1PR1*, *SMC1A*, *SOX2*, *STMN1*	Increased cell proliferation, migration, invasion, aerobic glycolysis, chemo-resistance, and EMT. Reduced apoptosis.	[27,92,94,95,96,97,98]
miR-29a	*CDC42*, *DNMT3A*, *DNMT3B*, *PDGFA*, *PDGFC*, *QKI-6*, *TRAF4*	Increased cell proliferation, migration, and invasion. Reduced apoptosis.	[99,100,101,102,104]
miR-30a	*BDNF*, *BECN1*	Increased chemo-resistance.	[105,106]
miR-34a	*BCL2*, *CDK6*, *MET*, *MSI1*, *NOTCH1*, *NOTCH2*, *PDGFRA*, *RICTOR*, *SIRT1*, *SMAD4*, *WNT6*, *YY1*	Increased cell survival, proliferation, invasion, and chemo-resistance. Reduced apoptosis.	[28,107,108,109,110,111]
miR-101-3p	*GSK3B*, *PTGS2*, *SOX9*, *TRIM44*	Increased cell proliferation, invasion, migration, metastasis, and chemo-resistance.	[114,115,116,117]
miR-124-3p	*AURKA*, *FOSL2*, *RHOG*, *SOS1*	Increased cell proliferation, invasion, and chemo-resistance. Reduced apoptosis.	[119,120,121,122]
miR-128-3p	*BMI1*, *E2F3*, *MET*, *PDGFRα*, *PTGS2*	Increased cell proliferation, migration, invasion, and chemo-resistance.	[124,125,126]
miR-142-3p	*AKT1*, *HMGA2*, *IL6*, *MGMT*, *RAC1*	Increased cell proliferation, migration, invasion, chemo-resistance, and immunosuppression.	[127,128,129,130]
miR-146a-5p	*NOTCH1*, *POU3F2*, *SMARCA5*	Increased cell survival, proliferation, and stemness.	[134,135,136]
miR-146b-5p	*EGFR*, *MMP16*, *TRAF6*	Increased cell proliferation, migration, and invasion.	[137,138,139,140,141]
miR-181a/b/c/d	*FBXO11* (miR-181a), *MGMT* (miR-181d), *RAP1B* (all), *SP1* (miR-181b)	Increased cell migration, invasion, and chemo-resistance.	[145,146,147,148]

**Table 3:** Circulating miRNAs in GB biofluid samples potentially serve as diagnostic and prognostic biomarkers.

Sample Type	miRNA(s)	Patient Cohort	Method of Detection	Use	Sensitivity/ Specificity	References
Blood	miR-128 and miR-342-3p	20 GB patients vs. 20 age- and sex-matched healthy controls	miRNA microarray and qPCR	Diagnosis	83%/79%	[149]
CSF	miR-10b and miR-21	19 GB patients vs. 15 patients with non-neoplastic neurological conditions	qPCR	Diagnosis	NA/NA	[150]
Cisternal CSF	miR-21-5p, miR-218-5p, miR-193b-3p, miR-331-3p, miR-548c-3p, miR-520f-3p, miR-27b-3p, miR-30b-3p, and miR-374a-5p	10 GB patients vs. 12 healthy controls	qPCR	Diagnosis	80%/67%	[151]
CSF-derived exosomes	miR-21	9 GB patients	qPCR	Diagnosis	NA/NA	[60]
Lumbar CSF	miR-21-5p, miR-218-5p, miR-193b-3p, miR-331-3p, miR-548c-3p, miR-520f-3p, miR-27b-3p, miR-30b-3p, and miR-374a-5p	18 GB patients vs. 20 healthy controls	qPCR	Diagnosis	28%/95%	[151]
Plasma	miR-7	3 untreated GB patients vs. 3 age- and sex-matched healthy controls	qPCR	Diagnosis	NA/NA	[25]
Plasma	miR-21, miR-128, and miRi-342-3p	50 GB patients vs. 10 healthy donors	qPCR	Diagnosis	90%/100%	[152]
Plasma	miR-454-3p	70 glioma patients vs. 70 healthy controls; 70 pre- vs. 70 postoperative glioma patients	qPCR	Diagnosis (glioma vs. healthy) and prognosis (pre- vs. postoperative)	NA/NA	[153]
Serum	miR-15b-3p, miR-23a, miR-133a, miR-150-3p, miR-197, miR-497, and miR-548b-5p	122 untreated high-grade astrocytoma (III-IV) patients vs. 123 healthy controls	Small RNAs sequencing and qPCR	Diagnosis	88%/97.87%	[154]
Serum	miR-17-5p, miR-125b, and miR-221	25 GB patients vs. 20 healthy controls	qPCR	Diagnosis	96%/100% for miR-17-5p; 95%/88% for miR-125b; 92%/100% for miR-221	[155]
Serum	miR-17-5p	324 treatment-responder GB patients vs. 302 non-responder GB patients	qPCR	Prognosis	50.5%/100%	[155]
Serum	miR-221	45 treatment-responder GB patients vs. 66 non-responder GB patients	qPCR	Prognosis	76.5%/100%	[155]
Serum-derived exosomes	miR-454-3p	Paired pre- vs. postoperative glioma patients	qPCR	Prognosis	NA/NA	[156]
Serum-derived exosomes	miR-454-3p	24 glioma patients vs. 24 age- and sex-matched healthy controls	qPCR	Diagnosis	79.17%/91.67%	[156]
Serum-derived exosomes	miR-210	91 glioma patients vs. 50 healthy controls	qPCR	Diagnosis	83.2%/95.3%	[157]
Tissue	miR-454-3p	24 glioma patients vs. 12 healthy controls	qPCR	Diagnosis	NA/NA	[156]
Tissue	miR-27a	19 GB patients vs. 7 surrounding non-tumor tissues	qPCR	Diagnosis	NA/NA	[158]
Tissue	miR-29a	147 astrocytic glioma vs. 20 non-tumoral controls	qPCR	Diagnosis	NA/NA	[100]

CSF: cerebrospinal fluid; NA: not available.

## Data Availability

Not applicable.
